# Supplementary material for: Flat Band and Hole-induced Ferromagnetism in a Novel Carbon Monolayer
Source: Sci Rep. 2019 Dec 27;9:20116. doi: 10.1038/s41598-019-56738-8 (PMC6934486; doi:10.1038/s41598-019-56738-8)
Supplement: Supplementary file 1 — Supplementary Information. [file 41598_2019_56738_MOESM1_ESM.pdf]

Supplemental Material for "Flat Band and Hole-induced Ferromagnetism in a Novel Carbon Monolayer"

Jing-Yang You,[†] Bo Gu,[*,‡,¶] and Gang Su[*,†,‡,¶]

School of Physical Sciences,University of Chinese Academy of Sciences, Beijing 100049, China, Kavli Institute for Theoretical Sciences, and CAS Center for Excellence in Topological Quantum Computation, University of Chinese Academy of Sciences, Beijing 100190, China, and Physical Science Laboratory, Huairou National Comprehensive Science Center, Beijing 101400, China

E-mail: gubo@ucas.ac.cn; gsu@ucas.ac.cn

In this supplemental material, we provide the result in checking the stability of cyclicgraphyne and cyclicgraphdiyne, density of states in hole-doped cyclicgraphdiyne, $k\cdot p$ models of doubly degenerate points in cyclicgraphdiyne, and tight-binding electronic and phonon flat bands for kagome lattice.

I. Stability of cyclicgraphyne and cyclicgraphdiyne

Figure S1 shows that cyclicgraphyne and cyclicgraphdiyne are kinetically and dynamically stable, and might be feasible in experiment.

[*]To whom correspondence should be addressed

[†]School of Physical Sciences,University of Chinese Academy of Sciences, Beijing 100049, China

[‡]Kavli Institute for Theoretical Sciences, and CAS Center for Excellence in Topological Quantum Computation, University of Chinese Academy of Sciences, Beijing 100190, China

[¶]Physical Science Laboratory, Huairou National Comprehensive Science Center, Beijing 101400, China

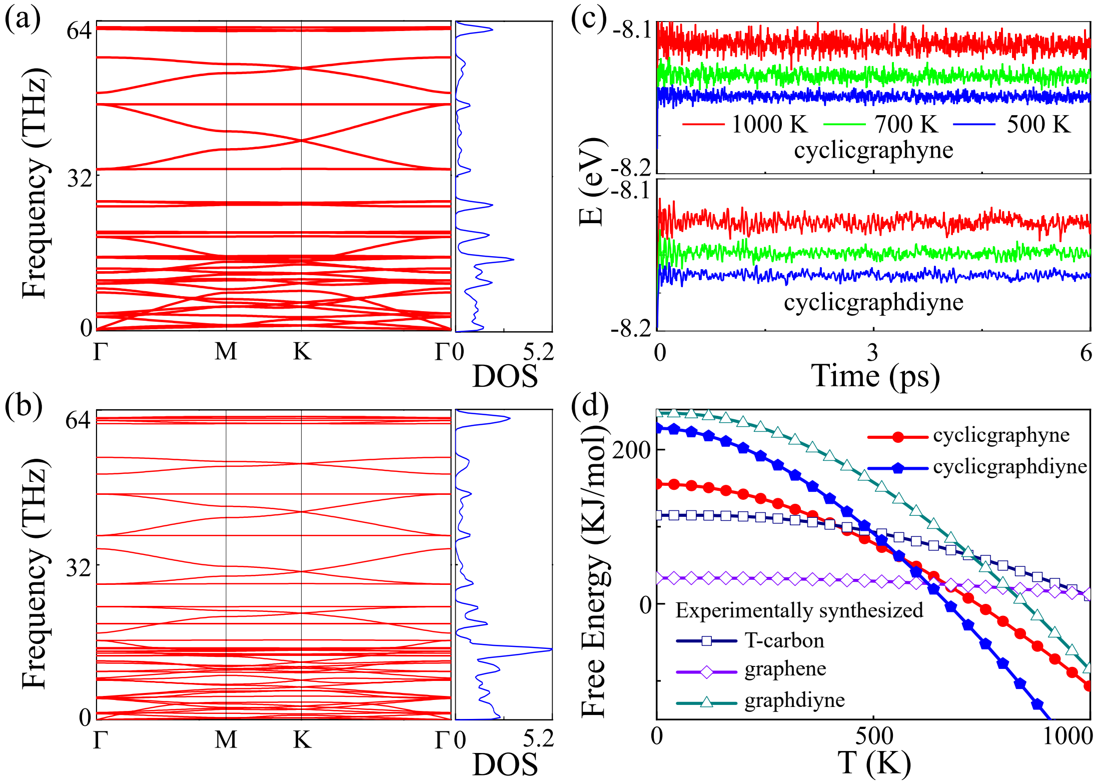

Figure S1: The phonon spectra and the corresponding density of states of (a) cyclicgraphyne, and (b) cyclicgraphdiyne. (c) For $3\times3\times1$ supercells of cyclicgraphyne and cyclicgraphdiyne, fluctuation of total energy per carbon atom as a function of time. The results are obtained by the molecular dynamics simulations at 1000 K (red line), 700 K (green line), and 500 K (blue line). (d) The free energies of cyclicgraphyne and cyclicgraphdiyne, and experimentally synthesized T-carbon, graphene and graphdiyne for a comparison.

II. Density of States in Hole-doped Cyclicgraphdiyne

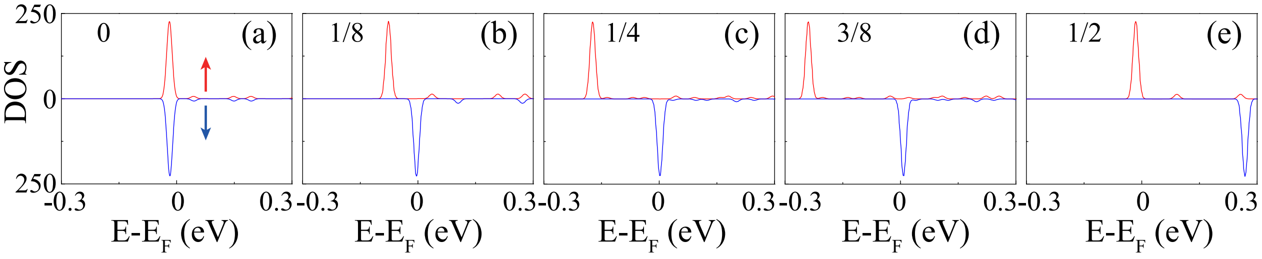

Figure S2: Density of states (DOS) in cyclicgraphdiyne (a) without doping, and with (b) 1/8, (c) 1/4, (d) 3/8 and (e) 1/2 hole concentration.

The DOS near Fermi energy with hole filling of 1/8, 1/4, 3/8 and 1/2 are plotted in Fig. S2. It is shown that the DOS at Fermi energy becomes finite with hole hoping, and becomes to nearly zero with half filling. This result shows the hole-doped cyclicgraphdiyne is a half-metal.

III. Doubly-Degenerate Points in Cyclicgraphdiyne

It is interesting to note that there exist doubly-degenerate point located at Γ and K points. We find that the two crossing bands at Γ point are a quadratic-type band and a flat band in all three directions, as shown in Fig. 2(c), while the two crossing bands at K points are of linear-type, i.e. Dirac fermions. To better understand the nature of quadratic dispersion as well as the low-energy quasiparticles, we construct $k \cdot p$ models for cyclicgraphdiyne at Γ and K points, respectively.

It is found that the symmetry at $K(K')$ point is of D_{3h}, and the irreducible representation of the crossing point at $K(K')$ point is E''. Using the two states as basis, one can construct the effective Hamiltonian constrained by the following symmetries: the three-fold rotation C_{3z}, the two-fold rotation C_{2x}, and the mirror symmetry M_z. Then, up to a linear order, we obtain the 2×2 effective Hamiltonian

$$\mathscr{H}(\boldsymbol{q}) = c + d(q_x\sigma_x - q_y\sigma_z), \tag{S1}$$

where $\boldsymbol{q}$ is measured from K. It is clear that the energy bands near K point are of linear-type (Weyl fermion).

The symmetry at Γ point is of D_{6h}, and we find that the two degenerate states at Γ point correspond to the 2D irreducible representation E_{1g}. Using the two states as basis, one can construct the effective Hamiltonian constrained by the following symmetries: the six-fold rotation C_{6z}, the two-fold rotation C_{2x}, the inversion $\mathscr{P}$, and the time reversal $\mathscr{T}$. Here, the space-time inversion $\mathscr{P}\mathscr{T}$ symmetry can be represented as $\mathscr{P}\mathscr{T} = \mathscr{K}$, with $\mathscr{K}$ the complex conjugation operator, which ensures that $\mathscr{H}(\boldsymbol{k})$ is real. Then, up to a quadratic order, we obtain the 2×2 effective Hamiltonian

$$\mathscr{H}(\boldsymbol{k}) = \varepsilon_0 \sigma_0 - 2bk_xk_y\sigma_x + b(k_x^2 - k_y^2)\sigma_z, \tag{S2}$$

where $\boldsymbol{k}$ is measured from Γ, $\varepsilon_0 = a_0 + a_1(k_x^2 + k_y^2)$, and $\sigma_{x,z}$ are Pauli matrices. The band dispersion at Γ point reads $E = a_0 + (a_1 \pm b)(k_x^2 + k_y^2)$. By fitting the DFT results in Fig. 2(c), the coefficients $a_0 = -0.011$ and $a_1 = b = 0.629$ are obtained. So, the crossing bands at Γ point are made from a quadratic band and a flat band. This fact might indicate that at Γ point there may exist a new type of two-fold fermion with simultaneous flat band and parabolic band.

IV. Tight-Binding Electronic Flat Band for Kagome Lattice

In order to better understand the origin of flat band contributed from p_z orbitals in cyclicgraphdiyne, we consider a 2D kagome lattice with carbon atoms as shown in Fig. S3(a) for a comparison, where the triangles in kagome lattice are corner-connected. The band structure of the 2D kagome lattice with carbon atoms is obtained by DFT calculations as shown in Fig. S3(b), where no flat band is observed for p_z orbitals. By using maximally-localized Wannier functions,[1] we obtain a TB model

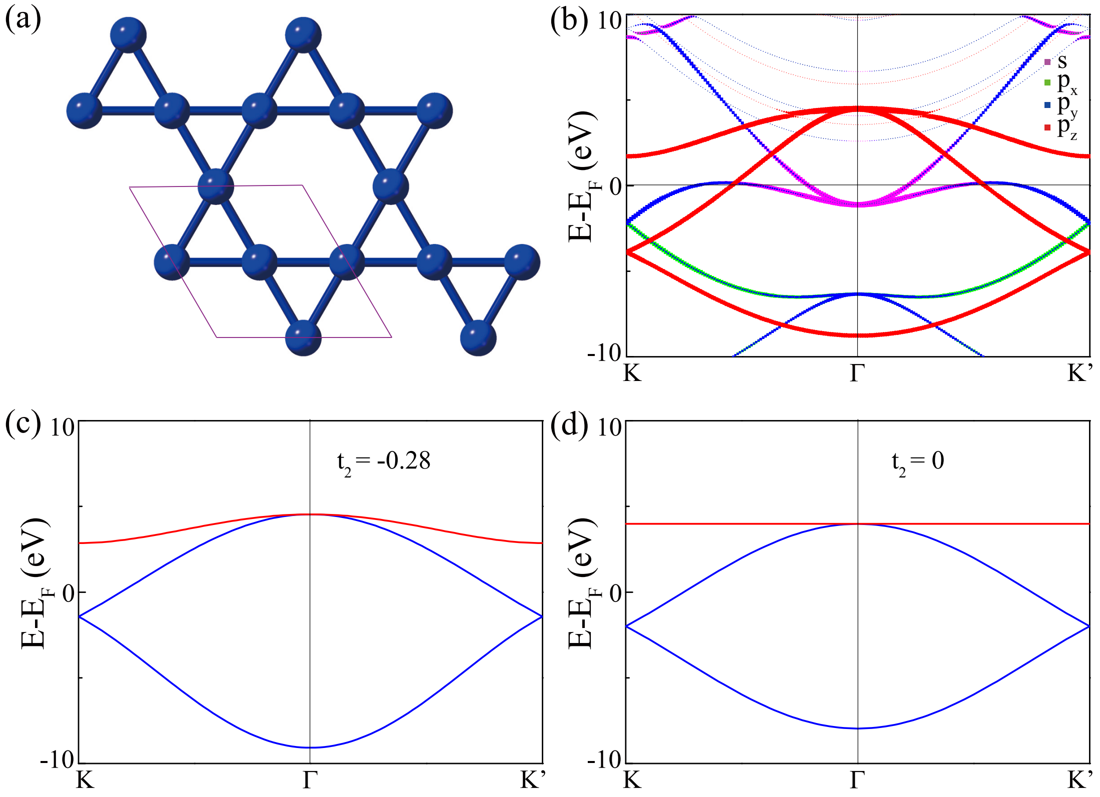

Figure S3: (a) The crystal structure of kagome lattice with carbon atoms, as well as the primitive cell in the purple box. Image created using VESTA 3.4.7 software (jp-minerals.org). (b) The flat bands of the kagome lattice. (c) The tight-binding bands based on three p_z orbitals in the primitive cell of the kagome lattice with hopping parameters $t_1 = -1.99$ and $t_2 = -0.28$. (d) The tight-binding bands with hopping parameters $t_1 = -1.99$ and $t_2 = 0$.

for p_z orbitals including the NN hopping and NNN hopping for the 2D kagome lattice

$$H = 2t_1 \begin{pmatrix} 0 & cos\vec{k}\cdot\vec{a}_1 & cos\vec{k}\cdot\vec{a}_2 \\ cos\vec{k}\cdot\vec{a}_1 & 0 & cos\vec{k}\cdot\vec{a}_3 \\ cos\vec{k}\cdot\vec{a}_2 & cos\vec{k}\cdot\vec{a}_3 & 0 \end{pmatrix} + \\ 2t_2 \begin{pmatrix} 0 & cos\vec{k}\cdot(\vec{a}_2+\vec{a}_3) & cos\vec{k}\cdot(\vec{a}_1-\vec{a}_3) \\ cos\vec{k}\cdot(\vec{a}_1+\vec{a}_2) & 0 & cos\vec{k}\cdot(\vec{a}_1+\vec{a}_2) \\ cos\vec{k}\cdot(\vec{a}_1-\vec{a}_3) & cos\vec{k}\cdot(\vec{a}_1+\vec{a}_2) & 0 \end{pmatrix}, \tag{S3}$$

where $\vec{a}_1 = (1,0)$, $\vec{a}_2 = (1/2, \sqrt{3}/2)$, $\vec{a}_3 = (-1/2, \sqrt{3}/2)$, t_1 and t_2 represent the NN and NNN hopping integrals, respectively. The NN and NNN hopping parameters $t_1 = -1.99$ and $t_2 = -0.28$ were obtained. The TB bands are shown in Fig. S3(c), which match well with the DFT bands in Fig. S3(b). When the NNN hopping is zero, a perfect flat band occurs with kagome lattice as shown in Fig. S3(d), which is consistent with the previous studies.[2–6]

V. Tight-Binding Phonon Flat Band for Kagome Lattice

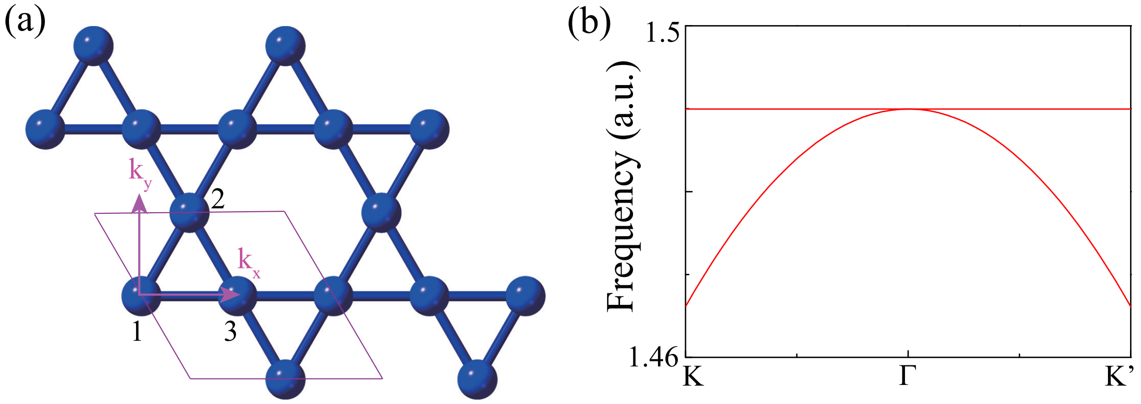

Figure S4: (a) The crystal structures of kagome lattice with atom index. Image created using VESTA 3.4.7 software (jp-minerals.org). (b) The tight-binding phonon flat band for kagome lattice.

In this section, we will give a detailed information about the origin of the phonon flat band. For

simplicity, we constructed the tight-binding model of phonons based on kagome lattice. The lattice vibration equation under the harmonic approximation can be obtained by $\widehat{M}\cdot\vec{A}=0$, where $\widehat{M}$ is the 3×3 matrix consisting of the frequency ω and dynamic matrix elements β, and $\vec{A}=[\vec{A}_1,\vec{A}_2,\vec{A}_3]^T$ and $\vec{A}_i$ represents the amplitude of wave for the i-th atom.

For kagome lattice with only the nearest-neighboring hopping, we have

$$\widehat{M}=\begin{pmatrix} D_1 & B_1 & C_1 \\ B_1^{\dagger} & D_2 & C_2 \\ C_1^{\dagger} & C_2^{\dagger} & D_3 \end{pmatrix}, \tag{S4}$$

where D$_1$=Diag$[m\omega^2-2(\beta_{1x,2x}+\beta_{1x,2y}+\beta_{1x,2z}+\beta_{1x,3x}+\beta_{1x,3y}+\beta_{1x,3z}), m\omega^2-2(\beta_{1y,2x}+\beta_{1y,2y}+\beta_{1y,2z}+\beta_{1y,3x}+\beta_{1y,3y}+\beta_{1y,3z}), m\omega^2-2(\beta_{1z,2x}+\beta_{1z,2y}+\beta_{1z,2z}+\beta_{1z,3x}+\beta_{1z,3y}+\beta_{1z,3z})]$, D$_2$=Diag$[m\omega^2-2(\beta_{2x,1x}+\beta_{2x,1y}+\beta_{2x,1z}+\beta_{2x,3x}+\beta_{2x,3y}+\beta_{2x,3z}), m\omega^2-2(\beta_{2y,1x}+\beta_{2y,1y}+\beta_{2y,1z}+\beta_{2y,3x}+\beta_{2y,3y}+\beta_{2y,3z}), m\omega^2-2(\beta_{2z,1x}+\beta_{2z,1y}+\beta_{2z,1z}+\beta_{2z,3x}+\beta_{2z,3y}+\beta_{2z,3z})]$, D$_3$=Diag$[m\omega^2-2(\beta_{3x,1x}+\beta_{3x,1y}+\beta_{3x,1z}+\beta_{3x,2x}+\beta_{3x,2y}+\beta_{3x,2z}), m\omega^2-2(\beta_{3y,1x}+\beta_{3y,1y}+\beta_{3y,1z}+\beta_{3y,2x}+\beta_{3y,2y}+\beta_{3y,2z}), m\omega^2-2(\beta_{3z,1x}+\beta_{3z,1y}+\beta_{3z,1z}+\beta_{3z,2x}+\beta_{3z,2y}+\beta_{3z,2z})]$,

$$B_1=2cos\frac{1}{2}(a_1+a_2)\cdot\begin{pmatrix} \beta_{1x,2x} & \beta_{1x,2y} & \beta_{1x,2z} \\ \beta_{1y,2x} & \beta_{1y,2y} & \beta_{1y,2z} \\ \beta_{1z,2x} & \beta_{1z,2y} & \beta_{1z,2z} \end{pmatrix},$$

$$C_1=2cos\frac{1}{2}a_1\cdot\begin{pmatrix} \beta_{1x,3x} & \beta_{1x,3y} & \beta_{1x,3z} \\ \beta_{1y,3x} & \beta_{1y,3y} & \beta_{1y,3z} \\ \beta_{1z,3x} & \beta_{1z,3y} & \beta_{1z,3z} \end{pmatrix},$$

$$C_2 = 2cos\frac{1}{2}a_2 \cdot \begin{pmatrix} \beta_{2x,3x} & \beta_{2x,3y} & \beta_{2x,3z} \\ \beta_{2y,3x} & \beta_{2y,3y} & \beta_{2y,3z} \\ \beta_{2z,3x} & \beta_{2z,3y} & \beta_{2z,3z} \end{pmatrix}.$$

Table S1: The dynamic matrix for kagome lattice. See Fig. S2(a) for the atom index 1, 2 ,3 and directions x, y, z.

β		1			2			3		
		x	y	z	x	y	z	x	y	z
1	x	0	0	0	6.36	-16.43	0	-4.58	10.11	0
	y	0	0	0	3.80	-0.93	0	-10.11	10.01	0
	z	0	0	0	0	0	0.37	0	0	0.37
2	x	6.36	3.80	0	0	0	0	6.36	-3.8	0
	y	-16.43	-0.93	0	0	0	0	16.43	-0.93	0
	z	0	0	0.37	0	0	0	0	0	0.37
3	x	-4.58	-10.11	0	6.36	16.43	0	0	0	0
	y	10.11	10.01	0	-3.80	-0.93	0	0	0	0
	z	0	0	0.37	0	0	0.37	0	0	0

The dynamic matrix elements can be obtained by using maximally-localized Wannier functions, and the results are listed in Table S1. The condition for the above equation to be solvable is that the determinant of coefficient matrix M equals to zero, i.e. Det(M)=0, leading to the phonon spectra. A plot [Fig. S4(b)] of the TB phonon spectra shows a flat band in optical branches. We can make the same conclusion for phonons as for electrons that the flat band comes from the NN hopping for kagome lattice. If we include the NNN hopping in the TB model on kagome lattice, we find that the flat band of phonons goes away and becomes dispersive.

VI. Flat Band and Hidden Valley Kagome Lattice in TBG

For TBG with small magic angles, the unit cell can be constructed from moiré pattern, which may be regarded as a huge triangular lattice, as depicted in Fig. S5, where the lattice points are comprised of the peaks of local density of states (LDOS) of TBG. However, if we look at the valleys

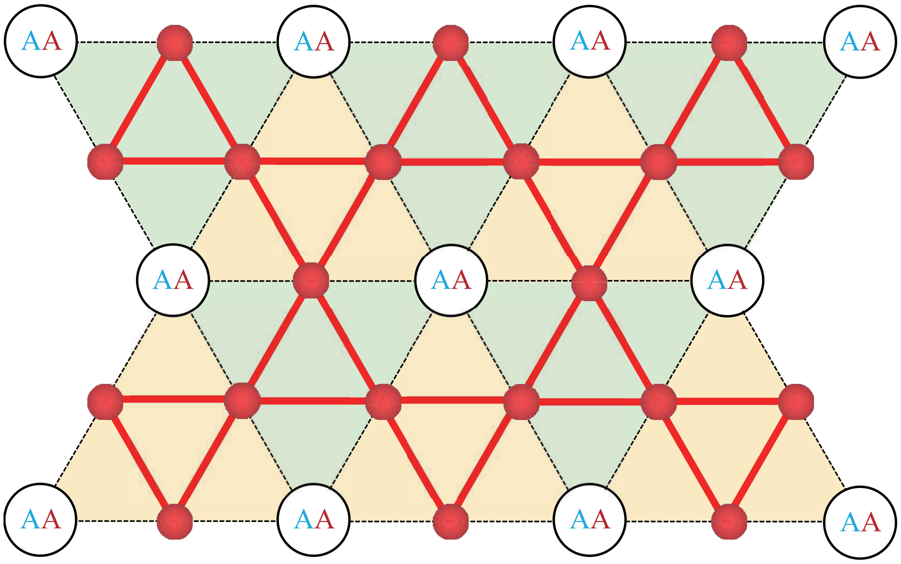

Figure S5: For TBG, the unit cell can be regarded as a huge triangular lattice, where the lattice points AA are comprised of the peaks of local density of states (LDOS) of TBG. By considering the valleys of LDOS, i.e., the center points of every neighboring AA points, one may observe that a kagome lattice is hidden in the TBG, as depicted by red lines.

of LDOS, i.e., the center points of every neighboring AA points, we find that a kagome lattice is hidden in TBG as shown in Fig. S5. The similarity between kagome lattice and hexagonal lattice was previously discussed.[2,3] It is known that the kagome lattice with only NN hopping can induce the flat band,[2,3] as we also addressed in the Supplemental Materials. In TBG, the hidden kagome lattice obtained from moiré pattern is quite large, so the NNN hopping should be much smaller than the NN hopping, and a nearly flat band can be obtained from such a hidden kagome moire lattice. This observation is also consistent with the flat band of cyclicgraphdiyne shown in Fig. 2(d), where the flat band mainly comes from the valleys of the total electron charge density.

There is a recent study also showing that a kagome structure exists in TBG, and a tight-binding model including both triangular and kagome lattices was proposed. In accordance with our present study, we believe that the flat band in TBG may be attributed to the hidden kagome lattice in moiré pattern.

References

(1) Marzari, N.; Vanderbilt, D. *Phys. Rev. B* **1997**, *56*, 12847–12865.

(2) Mielke, A. *J. Phys. A: Math. Gen.* **1992**, *25*, 4335–4345.

(3) Zhou, M.; Liu, Z.; Ming, W.; Wang, Z.; Liu, F. *Phys. Rev. Lett.* **2014**, *113*, 236802.

(4) Ye, L.; Kang, M.; Liu, J.; von Cube, F.; Wicker, C. R.; Suzuki, T.; Jozwiak, C.; Bostwick, A.; Rotenberg, E.; Bell, D. C.; Fu, L.; Comin, R.; Checkelsky, J. G. *Nature* **2018**, *555*, 638–642.

(5) Yin, J.-X. et al. *Nat. Phys.* **2019**, 1745–2481.

(6) Mizoguchi, T.; Udagawa, M. *Phys. Rev. B* **2019**, *99*, 235118.
